# ID 290 - Tecnovigilância dos Equipamentos Médico-Hospitalares no Brasil de 2014 a 2024

**DOI:** 10.5327/2237-9622.2025.v34s1.290

**Published:** 2025-11-25

**Authors:** Meline Rossetto Kron Rodrigues, Patrícia Sousa Silva Pinto, Natalliely Adriana da Luz, Cynthia Carolina Duarte Andrade

**Keywords:** equipamentos e provisões hospitalares, eventos adversos, tecnovigilância, segurança do paciente

## Abstract

**Introdução:**

Considerando o aumento significativo nos gastos com saúde e os recursos financeiros disponíveis limitados, torna-se relevante instituir estratégias de avaliação de desempenho dos produtos de saúde, como os equipamentos médico-hospitalares, e determinar os benefícios que essas tecnologias proporcionam para a saúde da sociedade. Este estudo objetiva descrever os dados brasileiros de tecnovigilância dos equipamentos médico-hospitalares no Brasil, de 2014 a 2024.

**Método:**

Consiste em um estudo descritivo sobre incidentes (eventos adversos) e queixas técnicas relacionados aos equipamentos médico-hospitalares notificados no Brasil de 12 de setembro de 2015 a 12 de setembro de 2024. Os dados foram coletados do Power BI da Agência Nacional de Vigilância Sanitária (Anvisa). Foram adotados os seguintes filtros: horizonte temporal de 12/9/2014 a 12/9/2024 e produto-motivo das notificações: equipamento médico hospitalar. Para facilitar a compreensão dos dados, estes foram assim apresentados: frequências (absoluta e relativa) das notificações (eventos adversos e das queixas técnicas); classificação destas quanto aos riscos associados ao uso dos equipamentos; e identificação dos equipamentos médico-hospitalares motivos, das não conformidades e de eventos adversos (conforme a nomenclatura adotada pela Organização Mundial da Saúde – OMS) mais ocorrentes.

**Resultados:**

Nos últimos dez anos, a Anvisa recebeu 16.981 notificações relacionadas a equipamento médico-hospitalar, sendo 8.026 (47,26%) referentes a eventos adversos e 8.955 (52,74%) relacionadas a queixas técnicas. Sobre os riscos associados ao uso dos equipamentos médicos-hospitalares, 66,63% (n=5.348) dos eventos adversos foram classificados como III (alto risco), 20,52% (n=1.838) das queixas técnicas foram classificadas como II (médio risco). Os equipamentos médico-hospitalares mais ocorrentes nas notificações foram bomba de infusão, com 3.181 (18,73%); sistema para cirurgia endoscópica, com 1.264 (7,44%); sistema para cirurgia oftalmológica, com 767 (4,52%); e foco cirúrgico, com 682 (4,02%). Entre as não conformidades informadas, outros (n=7.052; 41,53%) e não informado (n=3.308; 19,48%), não foram propriamente evidenciados os eventos adversos e as queixas técnicas mais ocorrentes. Em apenas 38,99% das notificações consta a identificação dos eventos adversos e da queixa técnica: quebra de material (n=527; 3,10%), elétrico/fonte de alimentação (n=445; 2,62%), problema da saída resultado incorreto ou inadequado (n=433; 2,55%).

**Conclusão:**

Considera-se que, quanto mais utilizada a tecnologia, maior a possibilidade de notificações reportadas à Anvisa, haja vista que, do total de notificações, 34,71% (n=5.894) referem-se a quatro equipamentos médico-hospitalares (bomba de infusão, sistema para cirurgia endoscópica, sistema para cirurgia oftalmológica e foco cirúrgico) que são muito utilizados nos diferentes serviços de saúde. Com relação às classes de risco dos equipamentos médico-hospitalares, espera-se que, quanto maior a eficiência de uma tecnologia, menor o risco associado ao seu uso. Nesse sentido, a maior parte dos incidentes que causam danos ao paciente (eventos adversos) é da classe III (alto risco). Entre as possíveis dificuldades relacionadas à identificação dos eventos adversos, destacam-se a adoção da nomenclatura padronizada pela OMS e a investigação da relação causal entre o incidente e o equipamento médico-hospitalar.

